# Craniofacial and Airway Morphology in Down Syndrome: A Cone Beam Computed Tomography Case Series Evaluation

**DOI:** 10.3390/jcm13133908

**Published:** 2024-07-03

**Authors:** Sonam Khurana, Ayman R. Khalifa, Nader N. Rezallah, Scott Lozanoff, Ahmed Z. Abdelkarim

**Affiliations:** 1 Department of Oral and Maxillofacial Pathology, Radiology and Medicine, NYU College of Dentistry, New York, NY 10010, USA; sonam.khurana@nyu.edu; 2 Department of Community Dentistry, College of Dentistry, Gulf Medical University, Ajman 4181, United Arab Emirates; dr.aymankhalifa@gmu.ac.ae; 3 Division of Oral and Maxillofacial Radiology, College of Dentistry, City University Ajman, Ajman P.O. Box 18484, United Arab Emirates; n.nabil@cu.ac.ae; 4 Department of Anatomy, Biochemistry & Physiology, University of Hawaii School of Medicine, Honolulu, HI 96813, USA; lozanoff@hawaii.edu; 5 Division of Oral and Maxillofacial Radiology, College of Dentistry, The Ohio State University, Columbus, OH 43210, USA

**Keywords:** Down syndrome, cone beam computed tomography, trisomy 21

## Abstract

**Background:** Down syndrome (DS) is a genetic condition characterized by an extra copy of chromosome 21, resulting in various physical and cognitive features. This study aimed to comprehensively analyze the dental and craniofacial morphology of individuals with DS using Cone Beam Computed Tomography (CBCT). **Methods:** Six individuals with DS, comprising five males and one female aged 17 to 35 years, underwent CBCT scanning. Radiographic assessments included dentition, occlusion, paranasal sinuses, airway, skull bones, and suture calcification. Linear and angular cephalometric measurements were performed, and airway analysis was conducted using Dolphin 3D imaging software v.11. **Results**: The study revealed prognathic maxilla in five patients, prognathic mandible in four, and bimaxillary protrusion in two. Dental findings included microdontia, enamel hypoplasia, and congenitally missing teeth, with maxillary and mandibular third molars most commonly absent. Sinus abnormalities, delayed suture closure, and cervical spine anomalies were also observed. **Conclusion:** These findings contribute to a deeper understanding of DS-related craniofacial characteristics and emphasize the importance of considering these morphometric features in clinical management strategies for individuals with DS. This study’s limited sample size underscores the significance of radiographic assessment in planning interventions such as cosmetic reconstructions, prosthetic rehabilitation, or orthodontic treatment for individuals with DS.

## 1. Introduction

Trisomy 21, also known as Down syndrome (DS), was initially identified by English physician John Langdon Down in 1866. Down used the term “individuals with Down syndrome (historically referred to as ‘Mongoloids’, a term now considered outdated and offensive)” to describe affected children, as they resembled people of Mongolian descent [1,2,3,4]. In 1956, Joe Hin Tjio and Albert Levan conducted groundbreaking research and discovered an extra copy of chromosome 21 in karyotype studies of patients [5,6].

Chromosomal non-disjunction during cell division is the primary cause of trisomy 21, which leads to three copies of the affected chromosome (95%). Approximately 3–4% of DS cases are attributed to chromosomal translocations, a type of familial DS, where a segment of chromosome 21 moves to another chromosome, usually chromosomes 14 or 15. The risk of passing translocated genes to offspring was higher for mothers who were carriers (12%) than for fathers (3%). As a result, the probability of DS increases in subsequent pregnancies when the child has a translocation. The third form of DS, known as mosaicism (<2%), occurs because of abnormal cell division following fertilization. This type of mosaicism is found among somatic cells of a similar type, leading to differential trisomy expression [5,7,8,9].

Down syndrome is a genetic condition that affects approximately 1 in 600–2000 live births in the United States [10] and is a common cause of intellectual disability of genetic origin [11]. The diagnostic features of this disease are complex and involve both mental and physical characteristics. Although a comprehensive phenotype of the disease can be demonstrated, unique craniofacial features play a crucial role in the diagnosis [12]. Recent studies [13,14,15] have employed two-dimensional (2D) and three-dimensional (3D) imaging modalities, such as lateral cephalometry, computed tomography, and Cone Beam Computed Tomography (CBCT), to examine the morphology of DS. Korayem and AlKofide (2017) used cephalometric radiographs to compare the craniofacial characteristics of individuals with DS to those of normal individuals and found that differences between the two groups could be observed when examining cephalometric radiographs [13]. Abeleira et al. (2015) used Cone Beam Computed Tomography (CBCT) images to study the hard palate in individuals with DS and reported that it was narrower in those with DS than in controls [14]. Additionally, the quantification of fluctuating dental asymmetry has been reported in individuals with DS [15], indicating that there are discrepancies in crown-to-root ratios and root length asymmetry, which are significantly lower in individuals with DS than in controls. Although few studies have focused on the craniofacial morphology of DS using Cone Beam Computed Tomography (CBCT) imaging [13,14,15], there is a lack of information regarding sutures, sinuses, and dental findings in the current literature. This study aimed to provide a comprehensive radiographic report that included an evaluation of common maxillofacial radiographic features, dental findings, cephalometric relationships, and airway characteristics in six individuals with DS assessed through Cone Beam Computed Tomography (CBCT) imaging.

## 2. Materials and Methods

### 2.1. Study Cohort

The research group comprised six individuals affected by DS (including one female and five male participants) aged between 17 and 35 years, with a mean age of 22.2 years. These six DS cases were the only ones found in the archive of the dental school and were matched with a control group of similar sex and age distribution. Specifically, the control group comprised six individuals without DS (including one female and five male participants) aged between 17 and 35 years, with a mean age of 24 years. The ages of the control group participants were 17 (male), 20 (male), 23 (male), 24 (male), 25 (female), and 35 (male). All participants underwent CBCT scanning. The inclusion criterion for this study was that none of the subjects had received previous orthodontic treatment or maxillofacial surgery.

**Figure 1:**
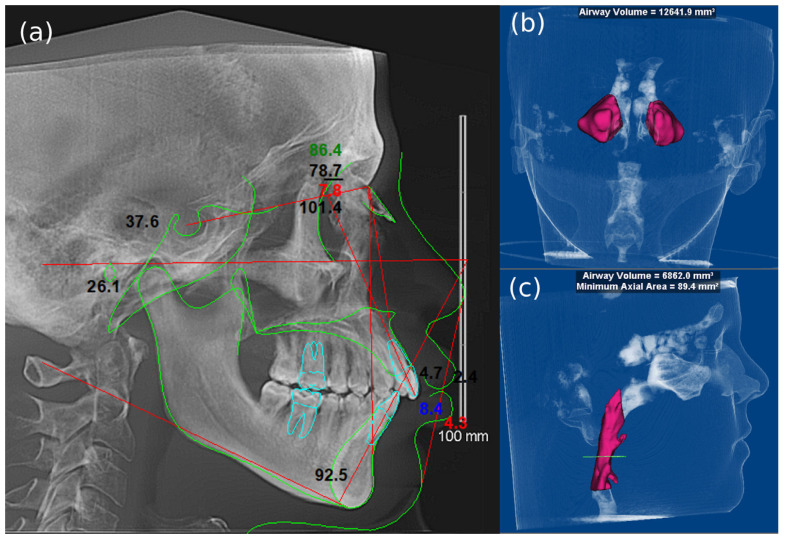
(**a**) A lateral cephalogram generated through cephalometric analysis, (**b**) a volumetric analysis of the bilateral maxillary sinuses, resulting in a computed volume of 12,641.9 mm^3^, and (**c**) a volumetric analysis of the pharyngeal airway, which revealed a total airway volume of 6862 mm^3^ and a minimum axial area of 89.4 mm^2^.

For the CBCT scans, a Planmeca CBCT machine (Promax 3D max; Planmeca, Helsinki, Finland) was utilized, featuring a 10 cm × 16 cm field of view (FOV) and voxel size of 0.4 mm. The DICOM files for each case were evaluated using OnDemand3D software version 1.0 (CyberMed Inc., Seoul, Korea). Two board-certified oral and maxillofacial radiologists and one board-certified orthodontist assessed the dentition, occlusion, paranasal sinuses, airway, skull bone, and suture calcification, and reported any incidental findings. Linear and angular cephalometric measurements were performed to provide diagnostic craniofacial analysis (Table 1 and Table 2: Figure 1a). Table 1 explains the definitions, and Table 2 explains the linear and angular cephalometric measurements. For the purpose of airway analysis, the CBCT images were obtained and imported into Dolphin 3D imaging software (version 11.5; Patterson Dental Supply, Chatsworth, CA, USA) on the same computer and monitor. The method used for airway measurements on CBCT images followed Glupker et al. (2015) [16], while volumetric measurements of the left and right maxillary sinuses and pharyngeal airway were added to the analysis parameters (Figure 1b,c).

**Figure 2:**
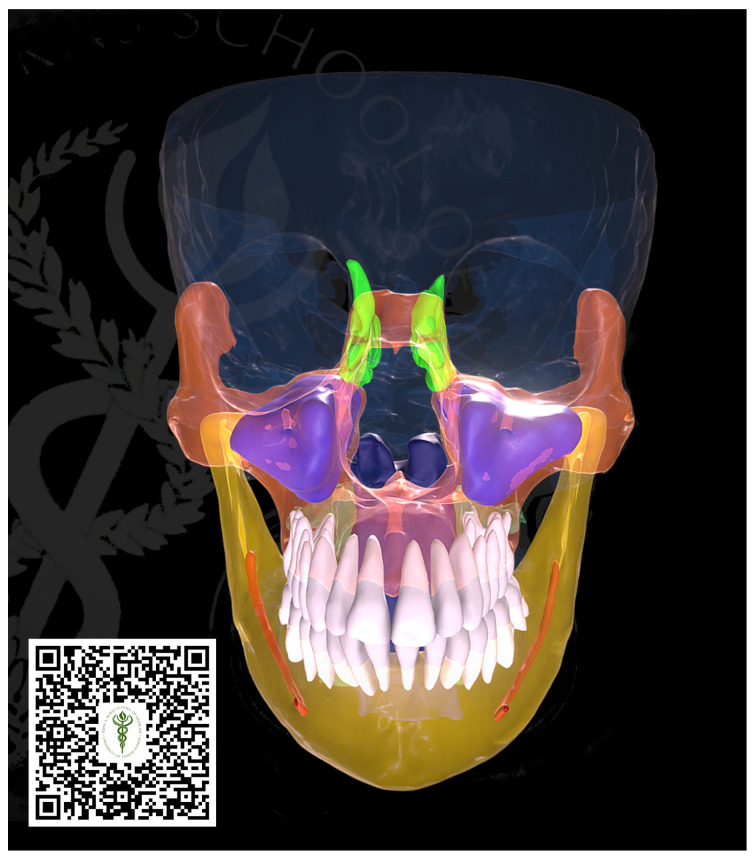
Three-dimensional surface model for visualization. The model presents some radiographic findings such as aplastic frontal and sphenoid sinuses and hypoplastic ethmoid sinuses. By scanning the barcode below using a phone camera, the 3D surface model can be manipulated on the phone screen.

The creation of surface models was intended to facilitate visualization and exchange. The pertinent structures were delineated using the software application 3D slicer (specific version, location). A range of threshold values was specified, and tools such as region growing, smart split, and Boolean operators were utilized to encapsulate the relevant structures. The polymesh segments were exported in the STL format and imported into a Pixologic Zbrush. Subsequently, the topologized models underwent retopologization by projecting the surface information from the original data onto the subdividing models, and a surface polishing algorithm was employed to retain landmarks while eliminating noise and faceting from the corresponding segmentations. Any areas with excessive noise or distortion were edited manually. Texture “baking” and texturing procedures were executed using the Adobe Substance Painter. The models and textures were exported and uploaded to Sketchfab (http://www.sketchfab.com), accessed on 7 February 2022, where additional material properties, lighting, and annotations were defined and applied. These were also integrated into Unity, where they could be viewed using various XR hardware systems, including Hololens, zSpace, and WebGL apps (see Figure 2).

### 2.2. Statistical Analysis

The information was documented and assessed through the application of Microsoft Excel version.16. Descriptive statistics, comprising means and standard deviations, were utilized to analyze the parameters. In cases where the distribution of the data was deemed to be normal, Student’s *t*-test for two independent samples was employed to compare the mean values of the quantitative variables. Conversely, when the distribution of the data was not deemed to be normal, the Mann–Whitney U-test was utilized to compare the mean ranks of the quantitative variables. The intraobserver variability was assessed by the same investigator at 2-week intervals, utilizing two of the six individuals and six controls. Statistical significance and precision of the estimates were determined using a *p*-value of less than 0.05 and 95% confidence intervals.

## 3. Results

### 3.1. Cephalometric and Airway Analysis

The correlation coefficient for intraobserver variability was greater than 0.80 for all values. The lateral cephalometric analysis for DS subjects is presented in Table 2 and Table 3. Out of the six patients, five had a prognathic maxilla with an average SNA angle of 89.38°, while one patient had an orthognathic maxilla with an SNA angle of 83.8°. On the other hand, four out of the six patients had a prognathic mandible with an average SNB angle of 86.3°, while two patients had an orthognathic mandible with an average SNB angle of 80.3°.

In six patients, five exhibited an inclination towards their upper incisors, with an average U1-SN angle of 113.48°. Only two patients displayed proclined lower incisors, accompanied by a mean L1-MP angle of 106.6°. Furthermore, two of the six patients showed bimaxillary protrusion. A comprehensive diagnostic summary derived from the lateral cephalometric analysis is presented in Table 3.

A formal presentation of the statistical analysis of the volume and surface area measurements between the DS group and its corresponding controls is provided in Table 4. The DS subjects displayed notably smaller nasal cavity and oropharynx volumes. Additionally, the minimum axial area at the level of the oropharynx exhibited a substantial discrepancy between the two groups, with a statistically significant difference observed.

### 3.2. Craniofacial Features

#### 3.2.1. Dental Findings

**Figure 3:**
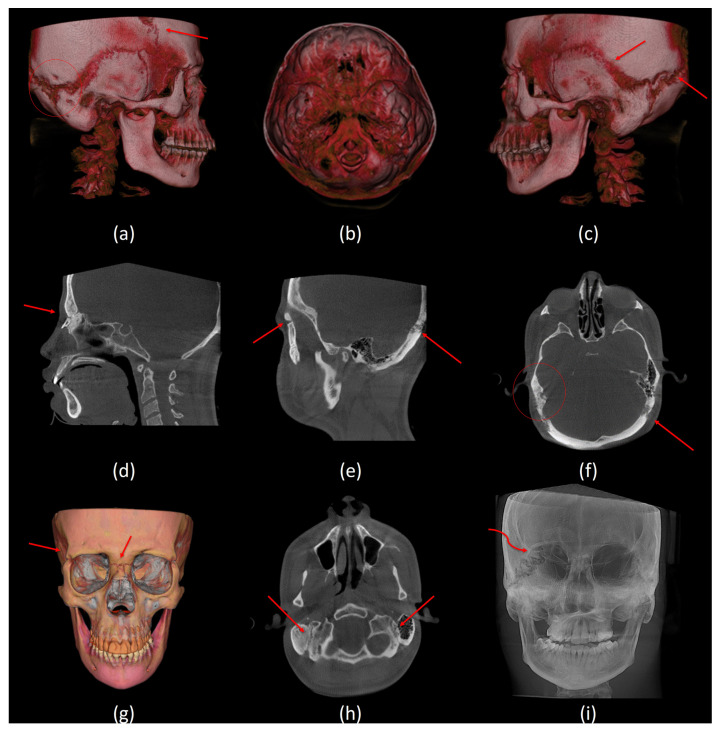
Sutures and other radiographic features. (**a**) A lateral view of 3D volumetric rendering shows delayed fusion of the coronal suture and Wormian bone at the occipital–mastoid region, (**b**) top view of 3D volumetric rendering shows copper beaten appearance, (**c**) a lateral view of 3D volumetric rendering shows delayed fusion of the squamous and lambdoid sutures, (**d**) a mid-sagittal CBCT slice shows delayed fusion of the frontonasal suture, (**e**) a sagittal CBCT slice shows delayed fusion of the frontozygomatic and lambdoid sutures, (**f**) an axial CBCT slice shows wormian bone on the right and delayed fusion of the lambdoid suture on the left, (**g**) a frontal view of 3D segmentation model shows delayed fusion of the right frontozygomatic suture and the frontonasal suture, (**h**) an axial CBCT slice shows delayed fusion of the occipitomastoid sutures, and (**i**) a frontal view of maximum intensity projection (MIP) shows delayed fusion of the right aspect of the lambdoid suture.

**Figure 4:**
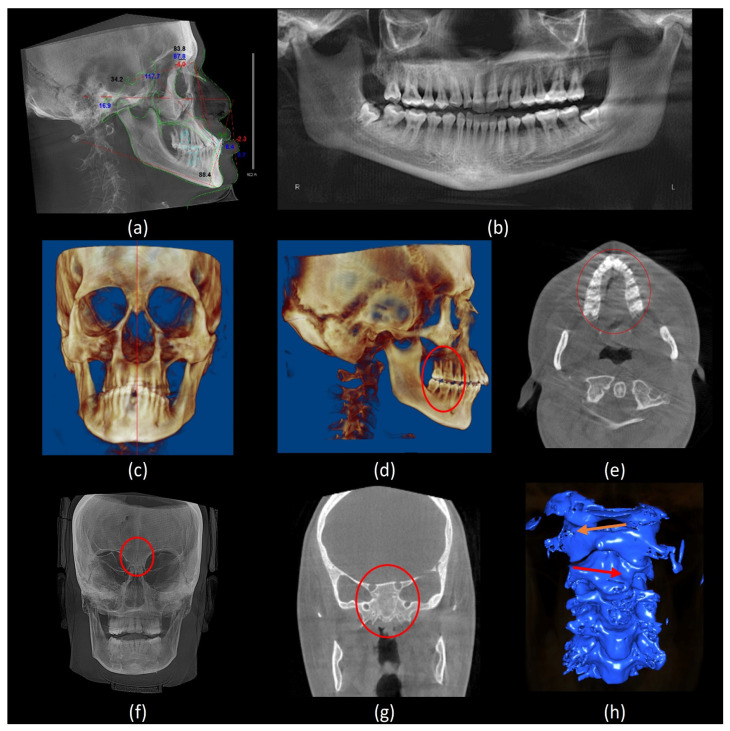
Various radiographic findings. (**a**) A lateral cephalometric analysis shows hypodivergent face type, hypoplastic maxilla, proclined anterior teeth, and anterior crossbite; (**b**) a panoramic reconstruction view shows a periodontal bone loss, impacted mandibular third molars and hypoplastic maxillary sinuses; (**c**) a 3D frontal volumetric rendering view shows facial asymmetry; (**d**) a 3D lateral volumetric rendering view shows Class II skeletal pattern (prognathic maxilla and orthognathic mandible) Molar class II full step (red circle); (**e**) an axial CBCT slice shows hypoplastic maxilla (red circle); (**f**) a frontal view of maximum intensity projection (MIP) shows aplastic frontal sinuses (red circle); (**g**) a coronal CBCT slice shows aplastic sphenoid sinuses (red circle); and (**h**) a 3D segmentation model of the cervical spine shows an anticlockwise-rotated C1 vertebra (orange arrow), accompanied by clockwise-rotated C2 and C3 vertebrae (red arrow).

For dental findings, the DS subjects share common features. Generalized enamel hypoplasia and microdontia were evident in most of them with subsequent teeth spacing in three out of six subjects. Anterior open bite and crossbite were seen in two subjects. In addition, posterior crossbite was evident in two subjects. All subjects presented with congenitally missing teeth, especially maxillary and mandibular third molars. However, only one subject had a retained deciduous tooth (#G). Periodontal bone level was normal in five subjects (Table 5). Figure 3 and Figure 4 depict various radiographic features of DS subjects. 

#### 3.2.2. Paranasal Sinuses, Sutures, and Cervical Spine

All subjects’ paranasal sinuses were evaluated. In most cases, the frontal sinuses were congenitally aplastic, although one subject showed hypoplastic frontal sinuses. The right and left maxillary sinuses were volumetrically smaller than controls in all subjects. Three subjects had aplastic sphenoid sinuses, while the rest showed hypoplastic sphenoid sinuses. Generally, the ethmoid sinuses appeared to be underdeveloped with fewer cells in all subjects. A granular bone pattern was observed at the aplastic sphenoid sinus site and some areas around the hypoplastic maxillary sinuses, consistent with arrested pneumatization in those areas.

Delayed fusion of the cranial sutures was a prominent characteristic in our sample. This was observed in the lambdoid, occipto-mastoid, squamous, coronal, fronto-zygomatic, and fronto-nasal sutures. Only one subject showed persistent spheno-occipital synchondrosis. Wormian bones were present in all six subjects. Four out of the six subjects had a brachycephalic skull shape with a thinned diploe. Cervical spine abnormalities, such as reduced intervertebral joint space and reduced atlantooccipital joint, were recorded in the DS group. One subject had an anticlockwise-rotated C1 vertebra, accompanied by clockwise-rotated C2 and C3 vertebrae, which represented the most severe anomaly in our sample (Figure 4h). Table 5 provides a summary of the dental and craniofacial features of the six DS cases using Cone Beam Computed Tomography (CBCT).

## 4. Discussion

This research examines the radiographic features of six individuals with Down syndrome (DS) using Cone Beam Computed Tomography (CBCT) scans. While previous studies have utilized CBCT to investigate aspects such as palate morphology, dental asymmetry, and orthodontic mini-screw placement [14,15,17], our study is distinct in its comprehensive reporting of dental and craniofacial morphology features for DS using large CBCT volumes. Furthermore, we provide in-depth insights into sutures and paranasal sinuses, enhancing the understanding of DS-related craniofacial characteristics.

Individuals with Down syndrome often display unique clinical characteristics that distinguish it from other craniofacial malformations. One such feature is microdontia, which affects a significant proportion of DS patients in both their primary and permanent dentition (35–55%) [18,19,20]. Early investigations by Kissling (1966) revealed that most teeth exhibited reduced crown size and complete root formation, with the exception of the upper first molars and lower incisors [21,22]. This unfavorable crown/root ratio often leads to tooth mobility and loss and morphological changes such as peg-shaped laterals and shovel incisors in the central and lateral incisors [23]. Furthermore, generalized spacing between teeth is commonly observed due to microdontia [24], a finding that was supported in our study, where four out of six subjects displayed microdontia, potentially resulting from a prognathic maxilla and mandible.

Furthermore, it is common for individuals with Down syndrome (DS) to experience hypoplasia and hypocalcification of enamel [24]. Hypoplasia, which can manifest as localized or generalized defects ranging from a smooth appearance to overt defects, is frequently associated with prolonged illness or fever [25]. In the cohort of subjects studied, all exhibited significant enamel hypoplasia. While enamel hypocalcification was not specifically assessed due to limitations in the clinical examination data available, teeth spacing emerged as a consistent observation within the study group. These findings contribute to a deeper understanding of the dental and craniofacial manifestations of DS and can inform more effective clinical management strategies for individuals with this syndrome.

All subjects displayed congenital absence of teeth, with the most commonly missing ones being the maxillary and mandibular third molars, along with teeth #6, #11, #23, and #26 in many cases. Partial anodontia occurs more frequently in individuals with Down syndrome (DS) at 50%, compared to only 2% in the general population [19]. Although the distribution of partial anodontia cases is similar between the two groups, this condition is inherited and often coexists with other ectodermal structural defects. The risk of partial anodontia significantly increases with a “trisomic insult” without targeting specific tooth buds [26]. The order of missing teeth, from most to least common, includes third molars, second premolars, lateral incisors, and mandibular incisors, with canines and first molars being rarely affected. Males are more commonly affected than females, with mandibular teeth being more affected than maxillary teeth and the left side more than the right [27]. Possible reasons for tooth agenesis include altered branching of the peripheral nervous system, resulting in fewer or stunted branches, and/or alterations in local cartilaginous tissue. The primary dentition of ten exhibits delayed exfoliation or retention. Although supernumerary primary teeth can occasionally be observed (~0.3% occurrence), they are less frequent than cases of partial anodontia. Maxillary arch crowding is more prevalent than mandibular arch crowding [25].

Taurodontism, which occurs in approximately 0.54% to 5.6% of individuals with Down syndrome (DS), is more prevalent in this population than in the general public [28]. Specifically, the mandibular second molar is the most commonly affected tooth. Taurodontism is caused by the failure of epithelial cells to migrate horizontally at the correct level and the slow proliferation of diaphragm cells, which are altered in DS [28]. It is noteworthy that our subjects did not display any cases of taurodontism.

The prevalent variation is characterized by small dimensions and an irregular crown shape due to mitotic delay in dental progenitor cells during embryonic development [29]. Typically, the labial surface and incisal edges of the anterior teeth, the cuspal inclines of the canines, the altered disto-lingual cusp of the maxillary molar teeth, and the displaced distal cusp of the mandibular molars [29] are the surfaces most commonly affected by irregular crown shape. It is important to note that our study did not assess crown morphological features due to the absence of clinical examination.

The incidence of caries is lower in the DS group compared to the general population [30]. Several factors contribute to this difference, including reduced Streptococcus mutans counts, delayed tooth eruption that decreases exposure to cariogenic environments, and higher pH and bicarbonate levels in saliva that increase its buffering capacity [31]. Additionally, congenital missing teeth and microdontia create open interproximal spaces, which reduces the incidence of caries. The presence of shallow fissures on occlusal surfaces also decreases the risk of caries [32]. Despite this, caries were present in all cases examined.

The eruption pattern of primary teeth in individuals with Down syndrome frequently varies from that of the general population. One of the most noticeable differences is the delay in the emergence of primary teeth, particularly the maxillary and mandibular anterior teeth and first molars. This delay usually begins around 12 to 14 months of age but can extend up to 24 months [29]. The completion of primary dentition eruption typically occurs around four to five years of age. In our study, only one case displayed a retained deciduous tooth (#G), which may be attributed to the advanced age of the participants, exceeding the typical timeline for deciduous dentition.

Concerning the eruption sequence of permanent teeth, individuals with Down syndrome generally adhere to a pattern similar to the general population, albeit with some delays. Typically, permanent teeth emerge without shedding primary teeth, with the upper and lower canines being the most commonly affected teeth. The eruption of molars and mandibular incisors is notably delayed until approximately 8 to 9 years of age [33].

In terms of occlusal abnormalities, individuals with DS commonly exhibit dental and skeletal malocclusions to a greater extent than the general population. These abnormalities include proclined upper and lower anterior teeth, anterior open bite, and anterior and/or posterior crossbite. In our study, three subjects presented with anterior open bite, while two exhibited bilateral posterior crossbite. This aligns with existing literature, which reports that approximately 50% of DS patients experience anterior open bite [21,26,27], although slightly lower frequencies, between 21% and 38%, are also reported [34,35]. Posterior open bite cases are much more variable in DS patients, with frequencies ranging from 33% to as high as 97% [21,33]. The findings from our study fall within this reported range and further support previous research.

Class III malocclusion is the most common occlusal pattern observed in individuals with Down syndrome (DS), seen in two patients in our study [10,18,36,37]. However, Cohen et al. (1970) reported different findings, wherein they identified Class I malocclusion as the most prevalent pattern in 47% of DS cases. On the other hand, Class II malocclusion is less frequently observed, ranging from 0 to 32% compared to Class I and Class III malocclusions [10,38]. In our sample, two out of five patients displayed Class I malocclusions.

DS patients exhibit a notable propensity for gingivitis compared to the general population despite similar levels of plaque accumulation. This heightened susceptibility to gingivitis can lead to significant periodontal issues, starting as early as 6 to 15 years of age. Early-onset periodontitis may progress to tooth mobility and extraction by the mid-30s [25]. Common periodontal problems associated with DS include marginal gingivitis, gingival recession, pocket formation, and horizontal and vertical bone loss with suppuration. Bifurcation and trifurcation involvement in the molar area, acute and subacute necrotizing gingivitis, advanced mobility, and loss of teeth, particularly in the mandibular anterior region, are also frequently observed [31,32]. Our study did not include the gingiva evaluation because patients were not evaluated clinically. Periodontal bone loss was present in only one patient.

In our cephalometric and airway analysis, we identified five patients with a prognathic maxilla, four with a prognathic mandible, and two with bimaxillary protrusion among the six individuals sampled. These findings contrast with the results reported by Korayem and AlKofide (2013) [13], whose study of sixty patients revealed a prevalence of retrognathic maxilla, prognathic mandible, and bimaxillary protrusion. The discrepancy in findings could be accounted for by differences in the population groups studied, with our study focusing on the Egyptian population, while their cohort consisted of Saudi Arabian individuals. Further exploration into dental features that may vary between the Egyptian and Saudi Arabian populations could provide additional insights into our interpretation of these divergent results.

Craniofacial anomalies are prevalent in individuals with Down syndrome (DS), encompassing microcephaly, brachycephaly, wide or open sutures, and a thin calvarium with partial or complete absence of diploe [39,40,41,42]. In our study, radiographic assessment revealed a consistent thinning of the diploe across all cranial regions in all subjects, consistent with observations made by Spitzer et al. (1961) and Frostad et al. (1971), who similarly noted a thin calvarium bone in DS patients [12,20]. Notably, the frontal and parietal bones are frequently affected, often exhibiting a nearly total absence of diploe formation [12]. Spitzer and Quilliam (1958) found that discrete metopic suture was the most common (75%) persistent suture in the DS group. Metopic suture closure was also delayed in 15% of the DS group with other cranial sutures. In the general population, delayed closure for all sutures was reported at 25% and 5%, respectively [43]. Delayed closure for all sutures was reported in all cases in Table 5.

Recognizing the prevalence and nature of craniofacial anomalies in individuals with Down syndrome (DS), such as aplastic and hypoplastic sinuses and maxillary hypoplasia, is crucial for gaining insight into the unique characteristics of our patient cohort. The extensive body of literature documenting these anomalies, including studies by Spitzer and Robinson [40], Spitzer and Quilliam [43], Spitzer et al. [20], and Frostad et al. [12], establishes a clear pattern of sinus underdevelopment and maxillary hypoplasia in DS cases. For example, Spitzer and Robinson (1955) reported a high incidence of aplastic and hypoplastic frontal sinuses and maxillary sinus abnormalities in DS cases [40]. Similarly, Spitzer et al. (1961) documented a significant prevalence of frontal sinus agenesis [20]. Our own observations align with these findings, as we identified hypoplastic maxillary sinuses and aplastic frontal and sphenoid sinuses in our subjects.

Moreover, the significance of maxillary hypoplasia has been emphasized by numerous studies, such as Alio et al. (2011) and Quintanilla et al. (2002), which have demonstrated its prevalence in DS cases [42,44]. This understanding enables us to anticipate and deal with the unique difficulties associated with maxillary growth and facial morphology in our patient group.

Furthermore, it is essential to understand associated anomalies, such as abnormalities in the shape of the sella turcica and atlantoaxial instability, in order to gain an in-depth understanding of the multisystemic nature of craniofacial abnormalities associated with Down syndrome [45,46]. This broader perspective is crucial for the holistic management of patients, as it enables the identification and intervention of potential complications related to these anomalies, such as cervical spine instability.

The detailed analysis of airway dimensions and sinus volumes in this study aligns with recent findings by Brown et al. [47], who emphasized the importance of volumetric analysis in diagnosing and managing airway obstructions in DS. Similarly, the study by Lee et al. [48] highlighted the role of advanced imaging in understanding the complex craniofacial morphology associated with DS, suggesting that these insights can inform more targeted therapeutic interventions.

In essence, the substantial body of literature on craniofacial anomalies in Down syndrome (DS) allows for a better understanding and management of the distinct clinical features and difficulties faced in our patient group, thereby facilitating diagnostic and therapeutic decisions that result in improved patient outcomes.

## 5. Limitations

The study’s limitations include a small sample size of six individuals with Down syndrome (DS), limiting generalizability. The homogeneous sample lacks ethnic and geographic diversity, and the age range of 17 to 35 years does not capture the full spectrum of craniofacial and dental issues across all ages. Using Cone Beam Computed Tomography (CBCT) for radiographic assessments, the study lacks comprehensive clinical data on enamel hypocalcification and crown morphology. It focuses on radiographic features, potentially overlooking functional outcomes and quality of life. Additionally, caries prevalence is based on radiographic evidence, which may be less accurate than clinical examinations, and there are no data on intervention outcomes like orthodontic treatments, prosthetic rehabilitation, and cosmetic reconstructions.

## 6. Conclusions

Our study examines craniofacial and dental features in individuals with Down syndrome (DS) using Cone Beam Computed Tomography (CBCT) scans. We found common dental anomalies such as microdontia, enamel hypoplasia, congenitally missing teeth, delayed eruption, and high caries prevalence. Craniofacial abnormalities included thin calvarium with absent diploe, hypoplastic or aplastic sinuses, delayed suture closure, and maxillary hypoplasia. Occlusal abnormalities consisted of prognathic maxilla and mandible, bimaxillary protrusion, anterior open bite, posterior crossbite, and prevalent Class III malocclusion. These findings are crucial for cosmetic reconstructions, prosthetic rehabilitation, and orthodontic treatment for DS patients, highlighting the importance of further research to enhance patient care.

## Figures and Tables

**Table 1:** The following table presents angular and linear cephalometric measurements, accompanied by their respective definitions.

Cephalometric Measurements	Definitions	
Skeletal	SNA angle	Angle between cranial base and sub-spinale (A-point)
	SNB angle	Angle between cranial base and supra-mentale (B-point)
	ANB angle	Difference between SNA and SNB
	SN-MP angle	Mandibular plane to cranial base angle
	FMA(MP-FH)	Mandibular plane to Frankfurt horizontal plane (orbitale–porion)
Dental	UI-NA	Upper incisor to nasion–A-point plane
	U1-SN	Upper incisor to sella–nasion plane
	LI-NB	Lower incisor to NB line (nasion–B-point)
	LI-MP	Lower incisors to mandibular plane (gonion–menton)
Soft tissue	LL-E Plane	Mean lower lip to Ricketts esthetic plane
	UL-E Plane	Mean upper lip to Ricketts esthetic plane

**Table 2:** The table below provides a comparison of angular and linear cephalometric measurements for individuals with Down syndrome.

Linear and Angular Cephalometric Measurements		Case 1		Case 2		Case 3		Case 4		Case 5		Case 6	
		Value	SD	Value	SD	Value	SD	Value	SD	Value	SD	Value	SD
Skeletal	SNA angle	90.5	3.5	86.4	3.5	88.0	3.5	88.0	3.5	94	3.5	83.8	3.5
	SNB angle	87.2	3.4	78.7	3.4	85.0	3.4	85.2	3.4	81.9	3.4	87.8	3.4
	ANB angle	3.4	1.5	7.8	1.5	3.0	1.5	2.8	1.5	12.2	1.5	−4.0	1.5
	SN-MP angle	26.8	5.2	37.6	5.2	33.2	5.2	35.3	5.2	31.2	5.2	34.2	5.2
	FMA(MP-FH)	14.8	4.5	26.1	4.5	20.9	4.5	25.5	4.5	19.9	4.5	16.9	4.5
Dental	UI-NA	6.9	2.7	4.7	2.7	5.5	2.7	1.4	2.7	0.0	2.7	8.0	2.7
	U1-SN	121.7	5.5	101.4	5.5	112.3	5.5	107.4	5.5	108.3	5.5	117.7	5.5
	LI-NB	9.6	1.8	8.4	1.8	5.0	1.8	7.2	1.8	8.5	1.8	8.4	1.8
	LI-MP	111.3	7.0	92.5	7.0	94.4	7.0	99.1	7.0	101.9	7.0	88.4	7.0
Soft tissue	LL-E Plane	0.2	2.0	4.3	2.0	−1.1	2.0	3.8	2.0	−2.0	2.0	3.7	2.0
	UL-E Plane	−2.2	2.0	2.4	2.0	−4.0	2.0	−1.2	2.0	−0.1	2.0	−2.3	2.0

**Table 3:** The table presents a summary of the lateral cephalometric diagnosis of Down syndrome cases. The data in the table are organized to present a clear and concise overview of the findings from the diagnosis. The table is an important tool for researchers and healthcare professionals to understand the characteristics of Down syndrome cases and develop effective treatment plans.

Case	1	2	3	4	5	6
Maxilla	Prognathic	Prognathic	Prognathic	Prognathic	Severely prognathic	Orthognathic
Mandible	Prognathic	Orthognathic	Prognathic	Prognathic	Orthognathic	Prognathic
Upper incisors	Proclined	Orthoclined	Proclined	Proclined	Proclined	Severely proclined
Lower incisors	Proclined	Orthoclined	Orthoclined	Orthoclined	Proclined	Orthoclined
Vertical Dimension	Hypodivergent	Within normal limit	Hypodivergent	Within normal limit	Hypodivergent—mouth opened	Hypodivergent
Occlusion	Class I molar relation	Class II molar relation	Class I molar relation	Class III dental occlusion	Cannot be assessed	Skeletal class III molar relation
Cant	Maxillary cant	No cant	No cant	No cant	No	Maxillary cant
Midline asymmetry	Deviated to left	Deviated to left	Midline on line	Deviated to right	Midline on line	Deviated to right
Dental midline	No deviation	No deviation	No deviation	No deviation	No deviation	Deviated to right
Periodontal bone	Generalized normal	Generalized normal	Generalized normal	Posterior bilateral upper and lower moderate periodontal bone loss	Generalized normal	Generalized normal

**Table 4:** The provided text presents a comparison between individuals with Down syndrome and controls in terms of airway analysis, along with relevant descriptive outcomes.

	Down	Control		
Measurements	Mean	Mean	*t*-Value	*p*-Value
Nasal Cavity (mm^3^)	12,748.65	24,098.83	4.18	0.001 ^1^
Nasopharynx (mm^3^)	3784.85	3753.26	0.033	0.97
Oropharynx (mm^3^)	9132.88	23,434.58	2.39	0.037 ^1^
Total (mm^3^)	12,735.57	27,191.77	2.17	0.055
MAA (mm^2^)	121.81	306.13	2.13	0.058
R Max Sinus (mm^3^)	6858.88	17,308.33	3.81	0.003 ^1^
L Max Sinus (mm^3^)	6087.6	18,473.08	4.56	0.001 ^1^

^1^ Significant at *p* ≤ 0.05.

**Table 5:** The table below summarizes the dental and craniofacial features of six previously unreported cases of Down syndrome (DS) using Cone Beam Computed Tomography (CBCT). The assessment of the scans was conducted by two oral and maxillofacial radiology residents, who compared the findings to those reported in the literature.

Comparison of Radiographic Features of Six Down Syndrome Patients						
	1	2	3	4	5	6
**Dental findings**						
Generalized enamel hypoplasia	Generalized thin enamel	Generalized thin enamel	Generalized thin enamel	Generalized thin enamel	Generalized thin enamel	Generalized thin enamel
Teeth size	Generalized microdontia	No	Generalized microdontia	Generalized microdontia	Generalized microdontia	No
Generalized spacing	Yes	No	No	Yes	Yes	No
Anterior open bite	Yes	No	No	Yes	Cannot be assessed due to open mouth position during scan acquisition	No
Crossbite	Bilateral posterior crossbite	No	Bilateral posterior crossbite	Anterior crossbite	Cannot be assessed due to open mouth position during scan acquisition	Anterior crossbite
Retained teeth	#G	No	No	No	No	No
Congenitally missing teeth	#1, #16, #17, and #32	#1, #16, #17, and #32	Missing #1, #16, and #17; #6 and #11; and #23 and #26	#1 and #16;	#1, #16, #17, and #32	#1, #16, #17, and #32
Impacted teeth	Partially impacted #11	No	No	No	No	No
Periodontal bone	Generalized normal	Generalized normal	Generalized normal	Posterior bilateral upper and lower moderate periodontal bone loss	Generalized normal	Generalized normal
**Paranasal Sinuses**						
Frontal sinus	Aplastic	Aplastic	Hypoplastic	Aplastic	Aplastic	Aplastic
Bilateral maxillary sinuses	Hypoplastic	Hypoplastic	Hypoplastic	Hypoplastic	Hypoplastic	Hypoplastic
Sphenoid sinus	Hypoplastic	Aplastic	Hypoplastic	Aplastic	Aplastic	Hypoplastic
Ostiomeatal complex	Patent	Patent	Not patent	Patent	Patent	Patent
**Sutures**						
Lambdoid and occipto-mastoid	Delayed fusion	Delayed fusion	Delayed fusion	Delayed fusion	Delayed fusion	Delayed fusion
Squamous	Delayed fusion	Delayed fusion	Delayed fusion	Normal	Delayed fusion	Delayed fusion
Coronal	Delayed fusion	Delayed fusion	Normal	Normal	Delayed fusion	Delayed fusion
Fronto-zygomatic sutures	Delayed fusion	Delayed fusion	Delayed fusion	Delayed fusion	Delayed fusion	Normal
Fronto-nasal suture	Delayed fusion	Delayed fusion	Delayed fusion	Normal	Delayed fusion	Normal
**Other radiographic features**						
Wormian bone	Yes	Yes	Yes	Yes	Yes	Yes
Skull shape	Brachycephalic	Normal	Brachycephalic	Normal	Brachycephalic	Normal
Cranium Diploe	Thinned Copper beaten	Thinned Copper beaten	Thinned Copper beaten	Thinned	Thinned Copper beaten	Thinned
C-spine	Hypoplastic shape	Reduced atlantooccipital joint space	Hypoplastic shape	Reduced atlantooccipital joint space	Reduced intervertebral joint space between C1 and C2	Anticlockwise rotation of C1 and clockwise rotation of C2 and C3
Basiocciput	Flattened	Flattened	Flattened	Flattened	Flattened	Flattened
High-arched palate	No	No	No	Yes	No	Yes
Bone pattern	Sphenoid and maxillary are granular	Normal	Normal	Sphenoid and maxillary bone is granular	Sphenoid and maxillary bone is granular	Normal
Pineal gland calcification	Yes	Yes	Yes	Yes	Yes	Yes
Other soft tissue calcifications	Bilateral choroid plexus	Bilateral choroid plexus	Bilateral choroid plexus			
Other findings					Patent spheno-occipital synchondrosis	

## Data Availability

The data presented in this study are available on request from the corresponding author. The data are not publicly available due to privacy.
